# *Gelidocalamus fengkaiensis* (Poaceae: Bambusoideae), a new bamboo species from Guangdong, China, with an analysis of branch development in relation to flowering

**DOI:** 10.1186/s40529-021-00319-4

**Published:** 2021-08-26

**Authors:** Zhuo-Yu Cai, Xin-Xin Zhou, Khoon-Meng Wong, Nian-He Xia

**Affiliations:** 1 Key Laboratory of Plant Resources, Conservation and Sustainable Utilization, Guangdong Provincial Key Laboratory of Digital Botanical Garden, South China Botanical Garden, Chinese Academy of Sciences, Guangzhou, 510650 China; 2 University of Chinese Academy of Sciences, Beijing, 100049 China; 3 Singapore Botanic Gardens, National Parks Board, 1 Cluny Road, Singapore, 259569 Singapore

**Keywords:** Morphology, taxonomy, Arundinarieae, branch complement, synflorescence

## Abstract

**Background:**

Bamboos, widely distributed in temperate and tropical Asia, Africa and America, refer to a group of special plants in Poaceae, Bambusoideae. China is rich in bamboo species. However, due to a long flowering cycle, the flowering habit and the flowering structure of many bamboo species are still not well understood. Here, we report a new bamboo species from Guangdong, China and an analysis of its interesting branch development in relation to flowering.

**Results:**

This species is similar to *G. stellatus*, the type species, but differs in the characteristics of its lemma and palea, mid-culm branch complement, and culm-sheath ligules. The initial branches at a culm node do not apically develop flowering structures during a flowering episode; instead, these form on what appears to be specialized flowering branches.

**Conclusions:**

The results of morphological comparison support the recognition of *Gelidocalamus fengkaiensis* as a new species. And during a flowering episode, two branch types (‘foliage branch’ and ‘flowering branch’) can be distinguished in this species.

## Background

*Gelidocalamus* T. H. Wen is a small genus classified into Arundinarieae tribe (Poaceae, Bambusoideae) with about 16 described species (Bamboo Phylogeny Group 2012; IPNI 2021; Wen 1982; Zhang et al. 2017). At first, this genus comprised only two species, *Gelidocalamus stellatus* T. H. Wen (type species) from Jiangxi province and *Gelidocalamus tessellatus* T. H. Wen & C. C. Chang from Guizhou province (Wen 1982).

For Guangdong province, Lin (1988, 1990, 1992) published three *Gelidocalamus* bamboos, namely *G. velutinus* W. T. Lin, *G. subsolidus* W. T. Lin & Z. J. Feng and *G. albopubescens* W. T. Lin & Z. J. Feng. Then, Xia (2005) also recorded *G. tessellatus* in Guangdong province. Xia and Lin (2009) included a total of four species in their *Flora of Guangdong* account. However, *G. albopubescens* was considered as synonym of *G. subsolidus* by Liu et al. (2017), because he reckoned these two species share the same vegetative morphological features including similar microscopic features of the abaxial leaf epidermis (Liu et al. 2017). Subsequently, Nie et al. (2018) reckoned that *G. stellatus* also occurs in Guangdong province.

During fieldwork in Qixingding Nature Reserve, Fengkai County, Guangdong province, a bamboo flowering in a few patches came to our attention. This species has leptomorph rhizomes, 3–5 branches per mid-culm node, just a single foliage leaf per ultimate branch, conventional spikelets, 3 stamens and 2 stigmas, which fall within the circumscription of *Gelidocalamus*. But after detailed examination of its vegetative and reproductive characters, we concluded that this bamboo is new to science, and is described and illustrated here. We also paid special attention to the development of the branch complement in relation to flowering.

## Methods

Flowering material was dissected under a stereo microscope (Mshot-MZ101) and images were taken with the camera attachment (Mshot-MSX2). Morphological comparisons were based on relevant literature (Liu et al. 2017; Lin 1988, 1990, 1992; Nie et al. 2018; Wen 1982), specimens and living plants. Both the type specimen and photos were used for making descriptions. The terms applied to the flowering structure in the analysis mainly follow the synflorescence concept applied to grasses (Cai and Xia 2021; Muchut et al. 2018; Reinheimer and Vegetii 2008; Stapleton 1997; Tivano et al. 2009; Vegetti and Müller-Doblies 2004). As the feature of branches bearing only a single foliage leaf is uncommon among bamboos, we specially examined and documented branch complements and axes at different stages of flowering to adduce their development from onset of flowering to senescence.

**Table 1:** Morphological comparisons of *Gelidocalamus fengkaiensis* and *G. stellatus*

Characters	*G. fengkaiensis*	*G. stellatus*
Foliage leaf size (cm)	15–29 × 4–6	12–17 × 1.3–2.2
Foliage leaf indumentum	Glabrous	Abaxially pubescent near midrib
Culm sheath margins	One margin densely ciliate, the other glabrous or apically ciliate	Glabrous
Culm sheath ligule	Pubescent	Glabrous
Mid-culm branches	3–5	7–12
Lemma apex	Mucronate	Aristulate
Palea apex	Acute	Bifid
Palea indumentum	Pubescent at the upper half between keels	Glabrous

## Results and discussion

### Elucidation of the new species

**Fig. 1:**
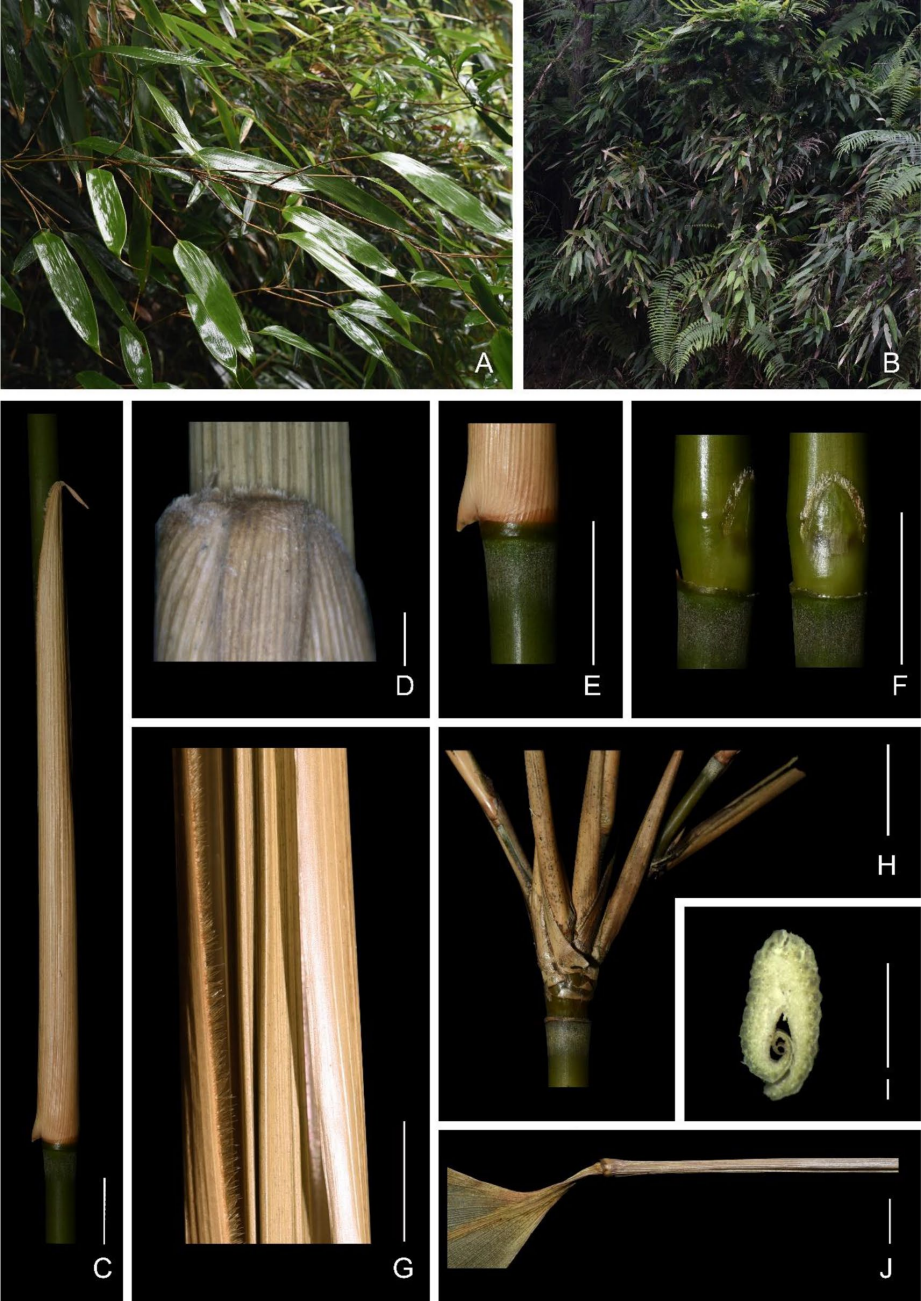
*Gelidocalamus fengkaiensis* N. H. Xia & Z. Y. Cai. **A**, **B** plant in the wild; **C** culm sheath; **D** apex of culm sheath; **E** pubescent zone below sheath scar, **F** primary bud; **G** culm sheath margin; **H** branches at mid-culm; **I** cross section of terminal leaf sheath; **J** terminal leaf sheath. Scale bars: **D** and **I** 1 mm; **C**, **J** and **E**–**H** 1 cm

***Gelidocalamus fengkaiensis*** **N. H. Xia & Z. Y. Cai, sp. nov. 封开短枝竹 (Figs.** 1 **and** 2 **).**

**Fig. 2:**
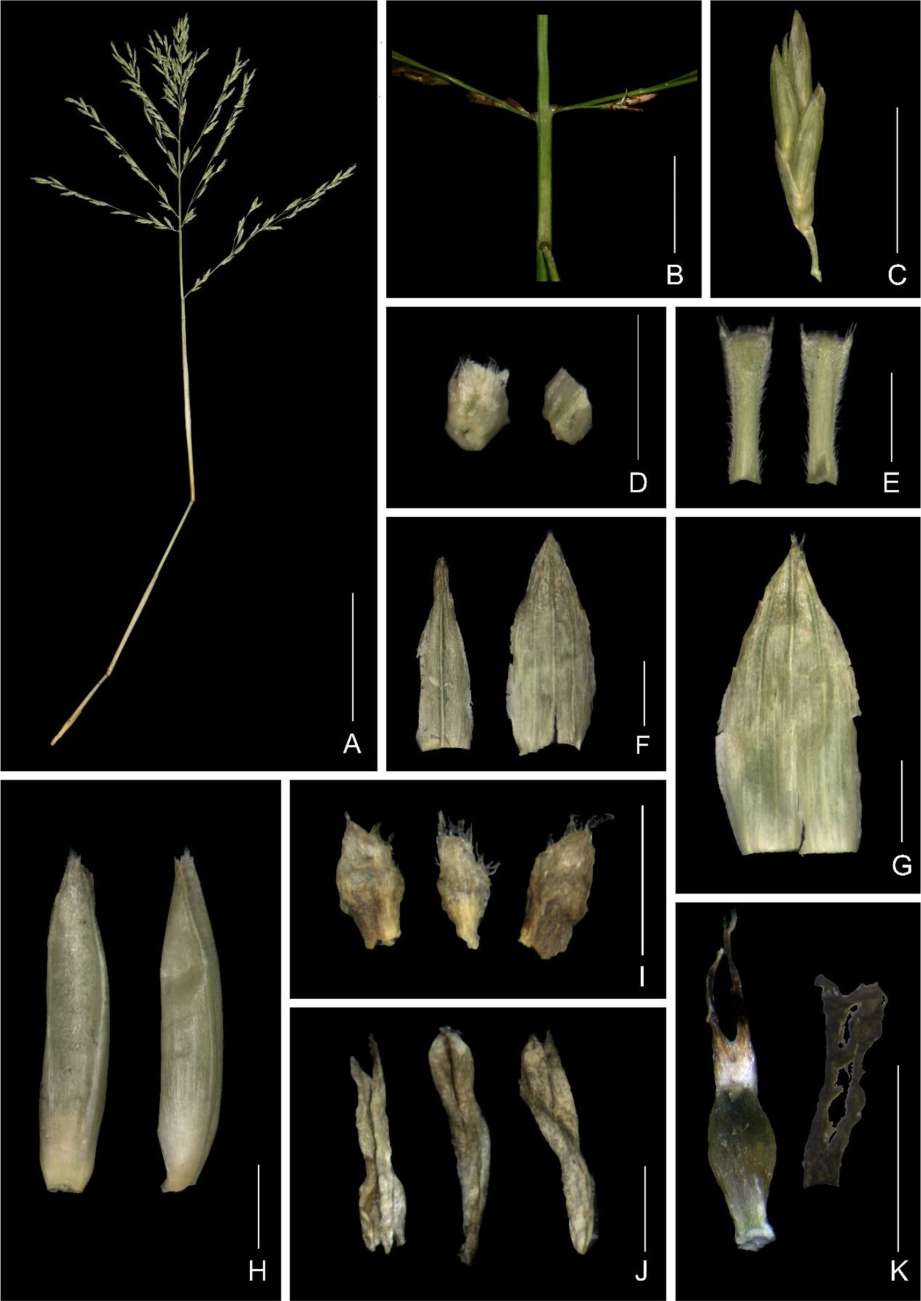
Floral structure of *Gelidocalamus fengkaiensis* N. H. Xia & Z. Y. Cai. **A** Flowering branch; **B** pulvini at the bases of long paracladia along inflorescence main axis; **C** spikelet; **D** callus, adaxial (left) and abaxial (right) view; **E** rachilla segment, adaxial (left) and abaxial (right) view; **F** first glume (left) and second glume (right); **G** lemma; **H** palea, adaxial (left) and side (right) view; **I** lodicules; **J** stamens; **K** ovary with styles (left) and stigmas with a part of style (right). Scale bar: **A** and **C** 5 mm; **B** and **D**–**K** 1 mm

**Type.** China, Guangdong, Fengkai County, He’erkou town, Qixingding Nature Reserve, Shenkeng village, under forest, roadside, 23° 31′ 59″ N, 111° 56′ 05″ E, 546 m, 3 August 2020, fl., Z. Y. Cai CZY-141 (holotype: IBSC! Barcode: 0865923, isotype: PE Barcode: 02352344).

**Diagnosis.***G. fengkaiensis* is similar to *G. stellatus*, but differs by its mucronate (versus aristulate) lemma apices, palea surfaces between keels that are pubescent (versus glabrous) at the upper half, acute (versus bifid) palea apices, 3–5 (versus 7–12) branches at mid-culm nodes, pubescent (versus glabrous) culm sheath ligules, culm sheaths with one margin densely ciliate and the other glabrous or apically ciliate (versus both margins glabrous), and larger foliage leaves of 15–29 × 4–6 cm (versus 12–17 × 1.3–2.2 cm) (Table 1).

**Description.** Culms 1–3 m tall; internodes initially green, 18–30 cm long, 5–8 mm diameter, glabrous, hollow; supranodal ridge prominent; sheath scar flat, with persistent remains of sheath base, and with a yellowish-brown pubescent zone below each sheath scar. Primary buds, solitary, ovate, compressed, the lateral edges densely ciliate except basally, apex acute, base rounded. Culm sheaths persistent, less than half as long as internodes, abaxial surface glabrous, one margin densely ciliate, the other margin glabrous or apically ciliate; auricles not developed; oral setae none or several, caducous; ligule subtruncate, ca. 1 mm high, densely pubescent; blade subulate or linear, abaxial surface sparsely pubescent, adaxial pubescent at base. Mid-culm nodes with 3–5 branches each, first-year (‘initial’) branches without higher-order branches; branch sheaths longer than internodes, abaxially glabrous, without black spots, marginal indumentum the same as culm sheaths. Foliage leaves 1 per ultimate branch, rarely 2; leaf sheaths thickened, tightly rolled, twig-like; leaf blades lanceolate to oblong, 15–29 cm long, 4–6 cm wide, glabrous, longitudinal veins (6–)7–8 pairs, base slightly truncate to cuneate, asymmetric, apex long-acuminate, one margin (that outer in the rolled up younger stage) serrulate, the other entire. The unit of inflorescence of the synflorescence panicle-like, (8.8–)11–13(–14) cm long; main axis glabrous or puberulent, basal internodes 1–5(–6) mm long, short paracladia 4–7, 1–4 mm long (excluding spikelets), each cladium bearing a single spikelet; primary long paracladia 9–13, 0.6–5.5 cm long (excluding spikelets), bases pulvinate, with several hairs or not, each cladium bearing 2–12 spikelets, longest cladium borne at the base of the main axis, secondary long paracladia present or not, when present, primary ones up to 8.6 cm long, bearing up to 29 spikelets. Spikelets 7–9 mm long; developed florets 3–4–(5), uppermost one not fully developed; rachilla segments compressed, ca. 1 mm long, puberulent except adaxial and abaxial surfaces glabrous below the middle; glumes 2; first (proximal) glume lanceolate to narrow-lanceolate, 2–3 mm long, glabrous, margin ciliate, 1–3-veined, apex acuminate; second glume lanceolate, 3–3.5 mm long, indumentum the same as the first glume, 3–5-veined, apex acute; lemma lanceolate, 3.5–4 mm long, glabrous, adaxial surface apically puberulent, margins ciliate or not, 5–7-veined, apex acute, mucronate, calluses puberulent except abaxial side; palea ca. 4 mm long, 2-keeled, apically pubescent and without veins between keels, each side glabrous and without veins, apex acute, comose; lodicules 3, ca. 8 mm long, middle one smaller, margins apically ciliate; stamens 3, ca. 3 mm long; styles 2; stigma 1 per style, plumose, ovary ellipsoid, ca. 1 mm long. Fruit unknown.

**Etymology.** The species epithet refers to the type locality, Fengkai county.

**Distribution and habitat.** This species is known only from the type locality, Qixingding Nature Reserve, Fengkai County, Guangdong Province, China. It commonly grows in secondary forest, under broad-leaved forest or by the roadside, at 500–600 m a.s.l. It is also found on the outskirts of villages.

**Additional specimen examined (paratype).** China, Guangdong Province, Fengkai County, He’erkou Town, Qixingding Nature Reserve, Lanchang village, road side, 23° 33′ 02″ N, 111° 56′ 33″ E, 513 m, 1 August 2020, Z. Y. Cai CZY-137 (IBSC!); Shenkeng village, 23° 31′ 59″ N, 111° 56′ 05″ E, 19 April 2020, fl., X. X. Zhou s.n. (IBSC!)

## Key to *Gelidocalamus* species in Guangdong

- Culms internodes glabrous—2.
- Culms internodes hairy—3.
- Mid-culm branches 7–12, foliage leaf size 12–17 × 1.3–2.2 cm —*G. stellatus*.
- Mid-culm branches 3–5, foliage leaf size 15–29 × 4–6 cm —*G. fengkaiensis*.
- Auricles of the culm sheaths conspicuous—*G. velutinus*.
- Auricles of the culm sheaths absent—4.
- Culms hollow, culm sheaths distally finely purple-brown checkered—*G. tessellatus*.
- Culms subsolid, culm sheaths not checkered—*G. subsolidus*.

## Branch development in relation to flowering

In *Gelidocalamus fengkaiensis*, the midculm branch complement (*sensu* McClure 1966) arises from a single primary bud. This bud produces a primary branch axis (subtended by a prophyll, Fig. 2F) that has a broad base but which soon continues to develop as a slender axis distally, at the same time developing several secondary branch axes from its basal nodes. This produces the basic branch complement of a cluster of several slender subequal (‘initial’) branches (i.e., of one primary and several secondary axes), with no obvious dominant member.

**Fig. 3:**
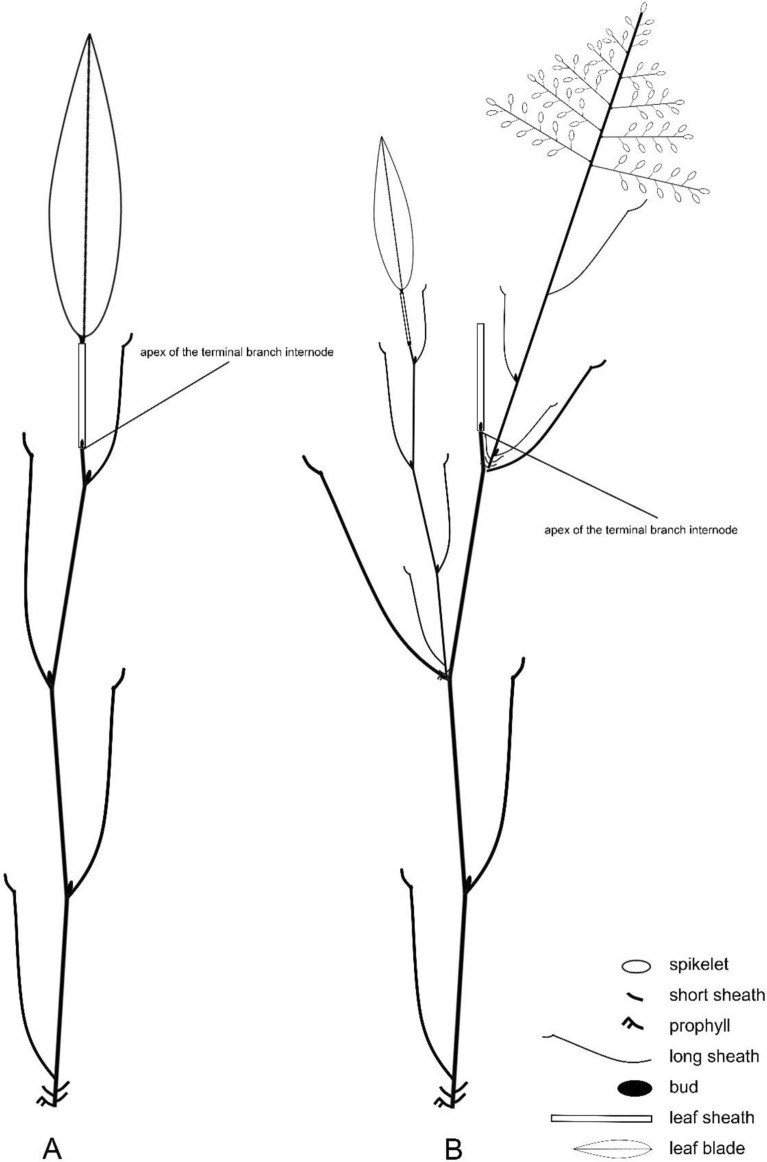
Diagram of components of foliage branches of *Gelidocalamus fengkaiensis* N. H. Xia & Z. Y. Cai. **A** Earlier phase foliage branch with leaf blade; **B** later phase foliage branch whose leaf blade has fallen off and some buds at branch nodes have developed as new axes. However, the apical meristem of the primary branch internode does not develop further

Each of these ‘initial’ branches in the basic branch complement develops a few closely spaced nodes basally but more distantly spaced nodes distally, characteristically giving rise to 3–4 elongate internodes (the primary branch axis itself sometimes developing up to five elongate internodes). Most of the branch nodes have buds and a number of these buds develop into similar vegetative branches of progressively higher order in subsequent growing seasons (Fig. 3B).

Two branch types can be distinguished in *G. fengkaiensis* as well as the generic type, *G. stellatus*. We call the branches, each of which terminates in a single foliage leaf, ‘foliage branches’ (= branches related to the production of leaves) (Figs. 1A, B, 3A, B). The more distal internodes on such foliage branches all have sheaths that are significantly longer than those at the branch base, but these also only have rudimentary blades, not expanded into the green ‘leaf blades’ that are typical of bamboo foliage leaf complements. In other species of *Gelidocalamus*, the distal-most two or several sheaths on a branch may also develop expanded green leaf blades (Zhu and Stapleton 2006). In *G. fengkaiensis*, typically, each foliage branch axis has a series of several well-spaced nodes but with only the distal-most node bearing the solitary expanded green leaf blade (Figs. 1A and 3A, B) (very exceptionally, 2-more such green leaf blades are borne). The final sheath of such a branch does not encase any elongate branch portion within, as revealed by sections of the distal-most sheath, but the whole resembles a twig due to its rigidity (Fig. 1I, J).

We also noted, from sections of the terminal leaf sheaths of foliage branches, that these were medially very much thickened and tightly rolled around the terminal meristem. Thus, in *Gelidocalamus*, the terminal branch sheath of foliage branches elongates far beyond the apical meristem and is especially thickened, and ideally could be expected to afford effective insulation against damage or drying out of the apical meristem.

In summary, the typical characteristics of *G. fengkaiensis* include a branch complement of several slender branches developed from a solitary primary branch bud; these initial branch axes individually bear only a terminal expanded foliage leaf blade (and are here referred to as foliage branches); and the distal-most leaf-sheath on such foliage branches far exceed the branch apex proper (i.e., apical meristem) and is especially thickened and tightly rolled, forming a rigid structure that supports the expanded terminal leaf blade.

The other branch type terminates in an inflorescence which we can refer to as a ‘flowering branch’ (Fig. 2A). As contrasted with foliage branches, flowering branches do not terminate in any expanded green leaf blades and appear to be specialized reproductive axes.

In the material of *G. fengkaiensis* we studied, the apical meristem (at the apex of the terminal branch internode) of a foliage branch appears to be hardly developed, even with the onset of flowering, so that this does not seem to elongate in time and appears to be dormant or even eventually senescent. We have found no evidence of such foliage branches (of primary or higher orders) continuing to develop a flowering axis. Apparently, during a flowering episode, flowering branch development is initiated by the available (axillary) buds at nodes on the primary and higher-order branch axes, i.e., buds that have not already developed earlier into foliage branches.

In *G. fengkaiensis*, we noted that whole-plant flowering seemed to be typical, but not all branch axes flowered simultaneously. Eventually, all branches, including both flowering branches or non-flowering foliage branches, became senescent and would perish. Flowering is supra-annual rather than annual. This would partly account for why to-date, *G. fengkaiensis* is only the fourth species of the genus for which we know the flowering structure. A flowering episode should last several months at least: we received information that this species was blooming in April, but when we went to collect it in August, flowering was clearly dwindling down and the culms were already starting to perish. The whole plant would die following flowering, essentially representing a monocarpic life history.

## Data Availability

Not applicable.

## Abbreviations

a.s.l.: About sea level
fl.: Flores (flowers)
IBSC: South China Botanical Garden, the Chinese Academy of Sciences, Herbarium
PE: Institute of Botany, the Chinese Academy of Sciences, Herbarium
s.n.: Sine numero (without number)
sp. nov.: Species nova (new species)
